# Supplementary material for: Prevalence of Tuberculosis in Central Asia and Southern Caucasus: A Systematic Literature Review
Source: Diagnostics (Basel). 2025 Sep 12;15(18):2314. doi: 10.3390/diagnostics15182314 (PMC12468312; doi:10.3390/diagnostics15182314)
Supplement: Supplementary file 1 [file diagnostics-15-02314-s001.zip › diagnostics-3833150-supplementary.pdf]

PREVALENCE OF TUBERCULOSIS IN CENTRAL ASIA AND SOUTHERN CAUCASUS: A SYSTEMATIC LITERATURE REVIEW

Supplementary file

Table S1. Prisma checklist

Section and Topic	Item #	Checklist item	Location where item is reported
**TITLE**			
Title	1	Identify the report as a systematic review.	On page 1
**ABSTRACT**			
Abstract	2	See the PRISMA 2020 for Abstracts checklist.	Supplementary file table 2
**INTRODUCTION**			
Rationale	3	Describe the rationale for the review in the context of existing knowledge.	On page 2
Objectives	4	Provide an explicit statement of the objective(s) or question(s) the review addresses.	On page 3
**METHODS**			
Eligibility criteria	5	Specify the inclusion and exclusion criteria for the review and how studies were grouped for the syntheses.	On page 4
Information sources	6	Specify all databases, registers, websites, organisations, reference lists and other sources searched or consulted to identify studies. Specify the date when each source was last searched or consulted.	Supplementary file table 5
Search strategy	7	Present the full search strategies for all databases, registers and websites, including any filters and limits used.	On page 3; Supplementary file table 4
Selection process	8	Specify the methods used to decide whether a study met the inclusion criteria of the review, including how many reviewers screened each record and each report retrieved, whether they worked independently, and if applicable, details of automation tools used in the process.	On page 4, 5
Data collection process	9	Specify the methods used to collect data from reports, including how many reviewers collected data from each report, whether they worked independently, any processes for obtaining or confirming data from study investigators, and if applicable, details of automation tools used in the process.	In this systematic review, data were collected from included reports using a standardized data extraction form. Two independent reviewers extracted data from each report to ensure accuracy and reduce bias. Any discrepancies between reviewers were resolved through discussion or by consulting a third reviewer.
Data items	10a	List and define all outcomes for which data were sought. Specify whether all results that were compatible with each outcome domain in each study were sought (e.g. for all measures, time points, analyses), and if not, the methods used to decide which results to collect.	The review focuses solely on the included studies' reported findings without actively extracting or synthesizing outcome data.
	10b	List and define all other variables for which data were sought (e.g. participant and intervention characteristics, funding sources). Describe any assumptions made about any missing or unclear information.	The review focuses solely on the included studies' reported findings without actively extracting or synthesizing outcome data.

Section and Topic	Item #	Checklist item	Location where item is reported
Study risk of bias assessment	11	Specify the methods used to assess risk of bias in the included studies, including details of the tool(s) used, how many reviewers assessed each study and whether they worked independently, and if applicable, details of automation tools used in the process.	Two independent reviewers extracted data from each report to ensure accuracy and reduce bias. Any discrepancies between reviewers were resolved through discussion or by consulting a third reviewer.
Effect measures	12	Specify for each outcome the effect measure(s) (e.g. risk ratio, mean difference) used in the synthesis or presentation of results.	The review is based solely on the reported findings of the included studies without statistical analysis or outcome measurement.
Synthesis methods	13a	Describe the processes used to decide which studies were eligible for each synthesis (e.g. tabulating the study intervention characteristics and comparing against the planned groups for each synthesis (item #5)).	The eligibility of studies for synthesis was assessed using the Newcastle-Ottawa Scale (NOS) to evaluate study quality. Studies were then grouped by country to facilitate comparisons across different healthcare settings and geographic regions. This approach allowed for a structured assessment of study relevance and quality before inclusion in the synthesis.
	13b	Describe any methods required to prepare the data for presentation or synthesis, such as handling of missing summary statistics, or data conversions.	The review relies solely on the qualitative assessment of the included studies.
	13c	Describe any methods used to tabulate or visually display results of individual studies and syntheses.	In this systematic review, results of individual studies were tabulated by grouping them based on country and study quality, as assessed using the Newcastle-Ottawa Scale (NOS). Additionally, a geographic map was created using Excel to visually display the distribution of studies across different regions.<br>We also compiled tables summarizing key data on prevalence, risk factors, and the economic burden reported in the included studies.
	13d	Describe any methods used to synthesize results and provide a rationale for the choice(s). If meta-analysis was performed, describe the model(s), method(s) to identify the presence and extent of statistical heterogeneity, and software package(s) used.	No meta-analysis was performed, and statistical heterogeneity was not assessed. The choice of narrative synthesis was made due to the heterogeneity in study designs, populations, and reported outcomes, which made quantitative pooling of results inappropriate.
	13e	Describe any methods used to explore possible causes of heterogeneity among study results (e.g. subgroup analysis, meta-regression).	Additionally, key findings related to prevalence, risk factors, and economic burden were summarized in tabular format to facilitate comparisons. A geographic map was created using Excel to visualize the distribution of studies across different regions. No subgroup analysis or meta-regression was conducted due to the diversity in study designs and reported outcomes.
	13f	Describe any sensitivity analyses conducted to assess robustness of the synthesized results.	No sensitivity analyses were conducted in this systematic review, as meta-analysis was not performed. The review relied on a narrative synthesis, grouping studies by country and study quality
Reporting bias assessment	14	Describe any methods used to assess risk of bias due to missing results in a synthesis (arising from reporting biases).	No formal methods were used to assess the risk of bias due to missing results in this systematic review, as meta-analysis was not performed.
Certainty assessment	15	Describe any methods used to assess certainty (or confidence) in the body of evidence for an outcome.	The review relied on a narrative synthesis, grouping studies by country and study quality
**RESULTS**			

Section and Topic	Item #	Checklist item	Location where item is reported
Study selection	16a	Describe the results of the search and selection process, from the number of records identified in the search to the number of studies included in the review, ideally using a flow diagram.	On page 5
	16b	Cite studies that might appear to meet the inclusion criteria, but which were excluded, and explain why they were excluded.	Supplementary file table 6
Study characteristics	17	Cite each included study and present its characteristics.	On page 5
Risk of bias in studies	18	Present assessments of risk of bias for each included study.	On page 4, table 1
Results of individual studies	19	For all outcomes, present, for each study: (a) summary statistics for each group (where appropriate) and (b) an effect estimate and its precision (e.g. confidence/credible interval), ideally using structured tables or plots.	On page 6 –11
Results of syntheses	20a	For each synthesis, briefly summarise the characteristics and risk of bias among contributing studies.	On page 6 –11
	20b	Present results of all statistical syntheses conducted. If meta-analysis was done, present for each the summary estimate and its precision (e.g. confidence/credible interval) and measures of statistical heterogeneity. If comparing groups, describe the direction of the effect.	On page 6 –11
	20c	Present results of all investigations of possible causes of heterogeneity among study results.	However, potential sources of heterogeneity were explored through narrative synthesis by grouping studies
	20d	Present results of all sensitivity analyses conducted to assess the robustness of the synthesized results.	not performed
Reporting biases	21	Present assessments of risk of bias due to missing results (arising from reporting biases) for each synthesis assessed.	not performed
Certainty of evidence	22	Present assessments of certainty (or confidence) in the body of evidence for each outcome assessed.	not performed
**DISCUSSION**			
Discussion	23a	Provide a general interpretation of the results in the context of other evidence.	On page 11
	23b	Discuss any limitations of the evidence included in the review.	On page 13
	23c	Discuss any limitations of the review processes used.	On page 13
	23d	Discuss implications of the results for practice, policy, and future research.	On page 14
**OTHER INFORMATION**			
Registration and protocol	24a	Provide registration information for the review, including register name and registration number, or state that the review was not registered.	PROSPERO 2025 CRD420251020764<br>Prevalence of tuberculosis in Central Asia and Southern Caucasus: a systematic literature review
	24b	Indicate where the review protocol can be accessed, or state that a protocol was not prepared.	https://www.crd.york.ac.uk/PROSPERO/view/CRD420251020764

Section and Topic	Item #	Checklist item	Location where item is reported
	24c	Describe and explain any amendments to information provided at registration or in the protocol.	
Support	25	Describe sources of financial or non-financial support for the review, and the role of the funders or sponsors in the review.	
Competing interests	26	Declare any competing interests of review authors.	
Availability of data, code and other materials	27	Report which of the following are publicly available and where they can be found: template data collection forms; data extracted from included studies; data used for all analyses; analytic code; any other materials used in the review.	

Table S2. – Prisma abstract checklist.

Section and Topic	Item #	Checklist item	Reported (Yes/No)
**TITLE**			
Title	1	Identify the report as a systematic review.	Yes
**BACKGROUND**			
Objectives	2	Provide an explicit statement of the main objective(s) or question(s) the review addresses.	Yes
**METHODS**			
Eligibility criteria	3	Specify the inclusion and exclusion criteria for the review.	Yes
Information sources	4	Specify the information sources (e.g. databases, registers) used to identify studies and the date when each was last searched.	Yes
Risk of bias	5	Specify the methods used to assess risk of bias in the included studies.	Yes
Synthesis of results	6	Specify the methods used to present and synthesise results.	NO
**RESULTS**			
Included studies	7	Give the total number of included studies and participants and summarise relevant characteristics of studies.	Yes
Synthesis of results	8	Present results for main outcomes, preferably indicating the number of included studies and participants for each. If meta-analysis was done, report the summary estimate and confidence/credible interval. If comparing groups, indicate the direction of the effect (i.e. which group is favoured).	Yes
**DISCUSSION**			
Limitations of evidence	9	Provide a brief summary of the limitations of the evidence included in the review (e.g. study risk of bias, inconsistency and imprecision).	Yes
Interpretation	10	Provide a general interpretation of the results and important implications.	Yes
**OTHER**			

Section and Topic	Item #	Checklist item	Reported (Yes/No)
Funding	11	Specify the primary source of funding for the review.	NO
Registration	12	Provide the register name and registration number.	

Impact Covid -19 Tuberculosis -TB Incidence During the Pandemic

Table S3 – FULL search strategy

Search limits	Studies in humans<br>English and Russian language<br>Full text available<br>Timeline: 2013 -2023
Data base	PubMed and Google Scholar
Disease terms	"Tuberculosis" OR "MDR-TB" OR "latent tuberculosis"
Country terms	"Kyrgyzstan" OR "Kazakhstan" OR "Mongolia" OR "Uzbekistan" OR "Tajikistan" OR "Turkmenistan" OR "Armenia" Or "Georgia" OR Azerbaijan"
Outcome terms	"burden" OR "economic" OR "prevalence" OR "incidence" OR "Covid -19 impact " OR " risk factors"
Combined	("Tuberculosis" OR "MDR-TB" OR "latent tuberculosis") AND ("Kyrgyzstan" OR "Kazakhstan" OR "Mongolia" OR "Uzbekistan" OR "Tajikistan" OR "Turkmenistan" OR "Armenia" Or "Georgia" OR Azerbaijan") AND ("burden" OR "economic" OR "prevalence" OR "healthcare costs" OR "costs" OR "expenditures" OR "spending" OR "YLD" OR "YLL" OR "DALY")

Table S4 – Search Results and Screening Process for Selected Databases

Search results		
1	***2***	***3***
Pubmed	1. Disease terms:	285,253
	2. Country terms:	33,828
	3. Outcome terms:	1,726,552
	4. Combined terms:	195
	5. Combined terms and published from 2018:	83
	6. Screening results:	151 full articles we tried to find full text for 12 we could not find in PubMed, from 12 we did not received the full text. All full text articles the total was 71
	7. Combined terms and full text available	71
	8. Article excluded:<br>- *Reasons*:	**60**;<br>wrong location of study: wrong outcomes =, wrong study design=, wrong setting =, wrong patient population =.
Google Scholar	1. Disease terms:	1 140 000
	2. Country terms:	178 000
	3. Outcome terms:	629 000
	4. Combined terms:	196

	5. Combined terms and published from 2018:	56
	6. Screening results:	56 full articles we tried to find full text for 13 we could not find Google, from 13 we did not received the full text. All full text articles the total was 43.
	7. Combined terms and full text available	43
	8. Article excluded:<br>- *Reasons*:	25<br>wrong location of study:<br>a. wrong outcomes =, wrong study design<br>b. =, wrong setting =, wrong patient population =.

Table S5 – Excluded Studies and Reasons for Exclusion

In this systematic review, the following studies appeared to meet the inclusion criteria but were **excluded** for specific reasons:

№	Author, Year	Excluded
1	Winetsky DE, 2014	Excluded due to insufficient data on key variables (**TB burden among prisoners**).
2	Truzyan N, 2020	Excluded because the study design did not align with the eligibility criteria (focused on service quality assessment rather than the required outcomes)
3	Kendall EA, 2015	Excluded as it did not provide country-specific data, making it difficult to compare with other studies in the synthesis
4	Skordis-Worrall J, 2017	Excluded due to high risk of bias, as assessed using the Newcastle-Ottawa Scale (NOS).

Table S6. Impact of the pandemic on TB in Central Asia

№	Country	Authors	Year of research	TB incidence after Covid-19
**1**	Kyrgyzstan	N.A.Altymysheva [47]	2023	In 2020, 3,518 cases of tuberculosis were registered, which is 31% lower than in 2019
**2**	Kazakhstan	Malika Gabdullina[49]	2018-2021	Comparing the pre-pandemic period with the pandemic period, there is a significant difference in the diagnosis of patients using routine screening (39% vs. 31%, $p < 0.001$), especially among those aged 60 years and older (16% vs. 22%, $p = 0.005$). During the pandemic, there is an increase in adverse outcomes from 11% to 20% (aRR = 1.83; 95% CI: 1.44–2.31), as well as an increase in case fatality from 6% to 9% ($p = 0.038$).
**3**	Tajikistan	USAID [50]	2020	In 2020, the number of reported cases of tuberculosis decreased by 28 percent compared to 2019, and the number of cases of drug-resistant tuberculosis (DR-TB) decreased by 34 percent.
